# Comparative analysis of proteomic profiles between endometrial caruncular and intercaruncular areas in ewes during the peri-implantation period

**DOI:** 10.1186/2049-1891-4-39

**Published:** 2013-10-05

**Authors:** Yang Wang, Chao Wang, Zhuocheng Hou, Kai Miao, Haichao Zhao, Rui Wang, Min Guo, Zhonghong Wu, Jianhui Tian, Lei An

**Affiliations:** 1 Ministry of Agriculture Key Laboratory of Animal Genetics, Breeding and Reproduction, National engineering laboratory for animal breeding, College of Animal Sciences and Technology, China Agricultural University, No.2 Yuanmingyuan Xi Lu, Haidian, Beijing 100193, China; 2 National Engineering Laboratory for Animal Breeding and MOA Key Laboratory of Animal Genetics and Breeding, China Agricultural University, Beijing 100193, China; 3 State Key Laboratory of Animal Nutrition, College of Animal Sciences and Technology, China Agricultural University, No.2 Yuanmingyuan Xi Lu, Haidian, Beijing 100193, China

**Keywords:** Caruncular areas, Endometrium, Ewe, Implantation, Intercaruncular areas

## Abstract

The endometrium of sheep consists of plenty of raised aglandular areas called caruncular (C), and intensely glandular intercaruncular areas (IC). In order to better understand the endometrium involved mechanisms of implantation, we used LC-MS/MS technique to profile the proteome of ovine endometrial C areas and IC areas separately during the peri-implantation period, and then compared the proteomic profiles between these two areas. We successfully detected 1740 and 1813 proteins in C areas and IC areas respectively. By comparing the proteome of these two areas, we found 170 differentially expressed proteins (DEPs) (*P* < 0.05), functional bioinformatics analysis showed these DEPs were mainly involved in growth and remodeling of endometrial tissue, cell adhesion and protein transport, and so on. Our study, for the first time, provided a proteomic reference for elucidating the differences between C and IC areas, as an integrated function unit respectively, during the peri-implantation period. The results could help us to better understand the implantation in the ewes. In addition, we established a relatively detailed protein database of ovine endometrium, which provide a unique reference for further studies.

## Background

Implantation, the sign and initial phase of pregnancy, is a process lading to attachment of developing conceptus to the maternal endometrium, and resulting in the establishment of placental structure. During the peri-implantation period, a synchronized and accurate crosstalk between conceptus and maternal endometrium, which is referred as maternal-fetal dialogue, must be established to support pregnancy [1]. It has been demonstrated that both the development of an embryo to the implantation-competent stage, as well as the transformation of the uterus into a receptive stage, are required for successful implantation [2]. Ovine has been extensively used as model to research maternal–fetal dialogue during implantation [3]. As an early sensor of embryos [4], endometrial functions are regulated primarily by progesterone (P4) from the corpus luteum, as well as cytokines and hormones secreted from the trophectoderm/chorion, including interferon tau (IFNT), to enter into a receptive status during early pregnancy [5]. In order to better understand the implantation process, detailed and comprehensive profiling of endometrium is necessary. However, the structure of endometrium in ruminants differs from other mammalian species Ovine uterine wall can be functionally divided into the endometrium and the myometrium. The normal adult ovine endometrium consists of LE, glandular epithelium (GE), several types of stroma (stratum compactum and stratum spongiosum), blood vessels and immune cells. In sheep, the endometrium has two distinct areas – aglandular caruncular (C) and glandular intercaruncular (IC). The C areas have LE and compact stroma and are the sites of superficial implantation and placentation [6], while the IC area, which is suffused with glandular epithelial cells [7,8], is mainly responsible for the synthesis and secretion histotroph, including enzymes, cytokines, growth factors, ions, hormones, glucose, transport proteins, and adhesion molecules to support early conceptus survival, development, implantation and placentation [2,9]. These two areas play different roles in implantation process, and both are essential for the establishment of pregnancy. Considering the significant structural and functional differences between C and IC areas, a comprehensive comparison between those two distinct endometrial areas could facilitate the understanding of endometrium involved implantations in ruminants.

Although implantation is of prime importance for establishment of pregnancy, the underlying mechanisms responsible for this complex physiological process, are still unclear. The high-throughput or ‘omic’ approaches, including RNA sequencing, microarray and proteome, has been applied to profile the expression patterns of genes or proteins for the endometrium during peri-implantation period. Walker et al. investigated the endometrial receptivity and maternal immunoregulation at day 17 of pregnancy in cattle using microarray [2]. Mansouri-Attia et al. recently profiled transcriptome of bovine endometrium during the peri-implantation period, after artificial insemination (AI) compared with the estrous cyclic endometrium using microarray, and found many factors and pathways essential to implantation in the C and IC areas [9]. Similarly, transcriptome of endometrium during early pregnancy have also been profiled in ewes [10], humans [11], and mice [12]. However, so far, most of these related studies were based on transcriptomic analysis. Compared to transcriptomic analysis, the proteomic approach has advantages of allowing people directly investigate functional molecules. Mullen et al. used label-free liquid chromatography-tandem mass spectrometry (LC-MS/MS) shotgun proteomics approach to characterize the uterine proteome at preimplantation stage in high fertility cattle [13]. Koch et al. first utilized LC-MS/MS proteomic technology to obtain the signature profile of proteins in the uterine lumen of ewes during early pregnancy [14]. However, in most of those studies, C and IC areas were not analyzed separately or compared mutually.

In the present study, we used LC-MS/MS technique to profile the proteome of endometrial C and IC areas separately at Day 17 of pregnancy, and established a relative detailed protein database of ovine endometrium during the time window of implantation. This database would provide a reference for future studies on implantation. By comparing the proteomic profiles between C areas and IC areas, we revealed the functional differences between these two areas during the critical period, which provided new information for studying the underlying molecular mechanisms responsible for structural and functional differentiation of these two areas. Finally, to our knowledge, this is the first report of endometrial proteome of C and IC areas, as an integrated tissue layer respectively, during peri-implantation in ewes.

## Materials and methods

### Animals and treatments

The experiments were performed were accordance with the Guide for the Care and Use of Agricultural Animals in Agricultural Research and Teaching, and all procedures were approved by the Institutional Animal Care and Use Committee at China Agricultural University (Beijing, China). Forty-six Chinese Small Tail Han ewes with normal estrus cycle were selected in our study. All ewes were fed and managed under a unified and optimized condition of environment and nutrition.

Forty-six cyclic ewes were estrus synchronized by using progesterone-impregnated (0.3 g) vaginal implants with controlled intra-vaginal drug release (CIDR-B™, Pfizer Animal Health, Auckland, New Zealand) for 13 d. Then each of these 46 ewes received 15 mg of prostaglandin F2α (Lutalyse, Pfizer, New York, NY, USA) intramuscularly 2 d before the progesterone vaginal implant was removed. Twenty-four h after removing the progesterone vaginal implant, three artificial insemination (AI) was performed within a 12-h interval. The day of the progesterone withdrawal was considered as day 0.

### Endometrial tissue recovery

All the ewes were slaughtered at Day 17 of pregnancy. Their uteri were flushed with PBS and collected. Ewes with the presence of normal elongated conceptus with a length of 25 cm or more attached to the endometrium were assigned pregnant and were sampled for proteomic analysis. Samples of the endometrial C and IC areas were taken and processed as described by Mansouri-Attia. The ipsilateral uterine horn was longitudinally opened by scissors. C areas were first carefully cut out then the IC areas were collected [4,9].

### Experimental design

**Figure 1:**
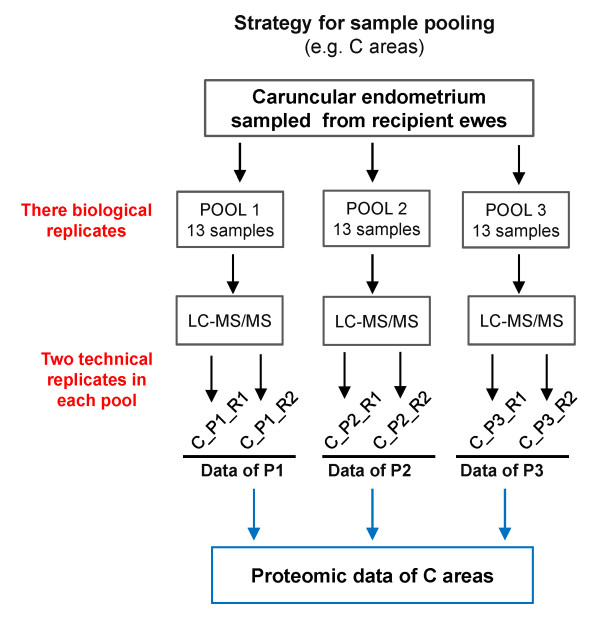
Experimental design of the study.

Given the significant structural and functional differences associated with C and IC areas, these two distinct endometrial zones need to analyze separately for a more comprehensive understanding of implantation in sheep. The global proteomic analysis of C and IC areas would provide new insights into the mechanisms responsible for implantation. Thus, endometrial C and IC areas were sampled and analyzed separately in the present study. For LC-MS/MS analysis, endometrial samples from ewes were divided into three pools for biological replicates, and each pooled sample was divided into two equal aliquots and processed as technical replicates (as shown in Figure 1). Data for each pool were obtained by averaging results from the two technical replicates.

### Protein extraction

All samples were ground to powder in liquid nitrogen and stored overnight at −20°C after adding a five-fold volume of chilled acetone containing 10% trichloroacetic acid (TCA) and 10 mmol/L dithiothreitol (DTT). Then the samples were centrifuged at 4°C, 16,000 × g for 20 min and the supernatant was discarded. The precipitates were added in 1 mL chilled acetone containing 10 mmol/L DTT before storing at −20°C for 30 min, then centrifuged at 4°C, 20,000 × g for 30 min. Centrifugation was repeated several times until the supernatant was colorless. The pellets were air-dried, then dissolved in lysis buffer containing 1 mmol/L phenylmethanesulfonyl fluoride (PMSF), 2 mmol/L ethylenediaminetetraacetic acid (EDTA) and 10 mmol/L DTT and sonicated at 200 Watts for 15 min before being centrifuged at 30,000 × g at room temperature for 30 min. The final protein concentration of supernatant was detected by using the Bradford method.

### Peptide digestion

Each sample taken 50 μg protein, then all isopycnic samples were formed by adding 8 mol/L urea solution. The samples were incubated with 10 mmol/L DTT at 56°C for 1 h to reduce disulfide bonds,and then added to 55 mmol/L iodoacetamide (IAM) in a dark room for 45 min to block cysteine bonding. Subsequently, each sample was diluted 8-fold with using 50 mmol/L ammonium bicarbonate and digested with Trypsin Gold at a protein:trypsin ratio of 20:1 at 37°C for 16 h. Final desalting using a Strata X C18 column (Phenomenex), the samples were vacuum dried. Peptides generated from digestion were directly loaded for LC-MS/MS analysis.

### LC-ESI-MS/MS analysis with LTQ-orbitrap collision induced dissociation (CID)

Each sample was resuspended in buffer A (2% acetonitrile (ACN), 0.1% formic acid (FA)) and centrifuged at 20,000 × g for 10 min. The final peptide concentration for each sample was about 0.5 μg/mL. The digested samples were fractionated using a Shimadzu LC-20 AD nano-high performance liquid chromatography (nano-HPLC) system. Sample loading (10 μL) was achieved using a 2-cm C18 trapping column in line with autosampler, and the peptides were eluted onto a resolving 10-cm analytical C18 column prepared in-house. The samples were loaded at a flow rate of 15 μL/ min for 4 min, and then a 91- min gradient from 2% to 35% buffer B (98% ACN, 0.1% FA) was run at a flow rate of 400 nL/ min, followed by a 5- min linear gradient to 80% buffer B that was maintained for 8 min before finally returning to 2% buffer B within 2 min. The peptides were subjected to nanoelectrospray ionization and then detected by MS/MS in an LTQ Orbitrap Velos (Thermo Fisher Scientific, Bremen, Germany) connected online to a HPLC system. The whole peptides were detected in the Orbitrap analyzer at a resolution of 60,000. Peptides were selected for MS/MS using the CID operating mode with a normalized collision energy setting of 35%, and ion fragments were detected in the LTQ. One MS scan followed by ten MS/MS scans was applied for the ten most abundant precursor ions above a threshold ion count of 5,000 in the MS survey scan. Dynamic exclusion was applied to increase dynamic range and maximize peptide identifications, and the parameters were set as follows: repeat counts = 2; repeat duration = 30 s; and exclusion duration = 120 s. The electrospray voltage was 1.5 kV. Automatic gain control (AGC) was used to prevent overfilling of the ion trap; 1 × 10^4^ ions were accumulated in the ion trap for generation of CID spectra. MS survey scans from m/z 350 to 2,000 Da.

### Proteomic analysis

Mass spectra were analyzed by using the MaxQuant software (version 1.1.1.36). As the genomic data of sheep is incomplete, we generated a reference protein database by integrating the following databases and sequences of cow proteins and current known sheep proteins and removed duplicate proteins, including GenBank nr (20110403), Uniprot cow proteins (20110503), sheep proteins [http://www.livestockgenomics.csiro.au/sheep/] and cow proteins [http://genomes.arc.georgetown.edu/drupal/bovine/]. The MS/MS data were searched against the reference protein database using the search engine embedded in MaxQuant. Up to two missed cleavages were allowed. The first search was set to 20 ppm, and the MS/MS tolerance for CID was set to 0.5 Da. To warrant the reliability and stability of our detection platform, following criteria for inclusion/ exclusion of peptides and proteins were used, as previous studies [15,16]: The false discovery rate (FDR) was set to 0.01 for peptide and protein identifications. Proteins were considered identified when at least two peptides were identified and at least one of which was uniquely assignable to the corresponding sequence. Contents of the protein table were filtered to eliminate identifications from the reverse database and common contaminants. In the case of identified peptides that were all shared between two proteins, these were combined and reported as one protein group. Contents of the protein table were filtered to eliminate identifications from the reverse database and common contaminants. The minimum peptide length was set to 6 amino acids. A minimum of two peptides with one being unique was required for protein identification. To perform label-free quantification analysis, the MaxQuant software suite containing an algorithm based on the extracted ion currents (XICs) of the peptides was used. Xcalibur 2.1 (Thermo Scientific) was used as quality control program to check the quality of chromatographs.

### Data analysis

In data analysis, all proteins were mapped to the Ensembl Bos taurus gene ID. The expression quantity of each protein normalized on the basis of the numbers of peptides by using MaxQuant software (version 1.1.1.36). Only peptides which is corresponding to unique proteins could use to protein quantization in the comparison between peptides and proteins. In the comparison of proteomic profiles between C and IC areas, the measured value of each biological replicate was achieved by averaging every two technical replicates of a biological replicate. Then the Student’s t-test was used to detect the significance of the differentially expressed proteins (DEPs) according to the measured value of every three biological replicates in each group, and *P* < 0.05 was considered significant.

We used DAVID (The Database for Annotation, Visualization and Integrated Discovery) version 6.7 platform [http://david.abcc.ncifcrf.gov/] annotate biological themes for DEPs between C and IC areas. This platform often used to analyze high-throughput data [17,18].

To assess the similarities of the different replicates, and to obtain a visual understanding of the relationship between the different areas, hierarchical clustering was carried out using CLUSTER 3.0 data analysis tool based on the clusters of protein expression profile of different technical and biological replicates.

## Results

### Summary of the endometrial proteome in C and IC areas

**Figure 2:**
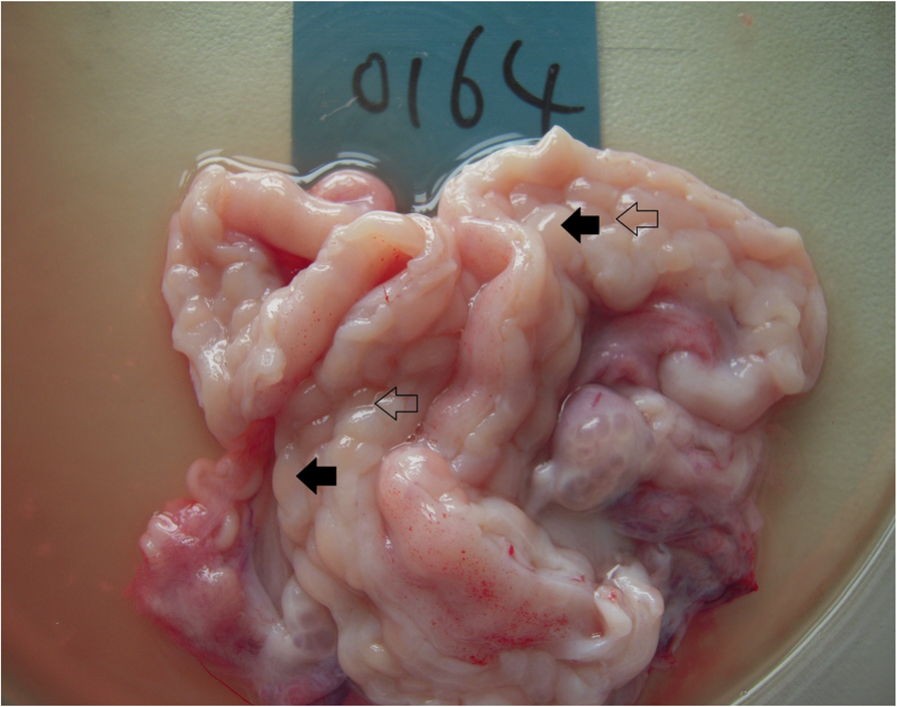
**The endometrium of Chinese Small Tail Han ewes.** Black arrows pointed to C areas, white arrows pointed to IC areas.

**Figure 3:**
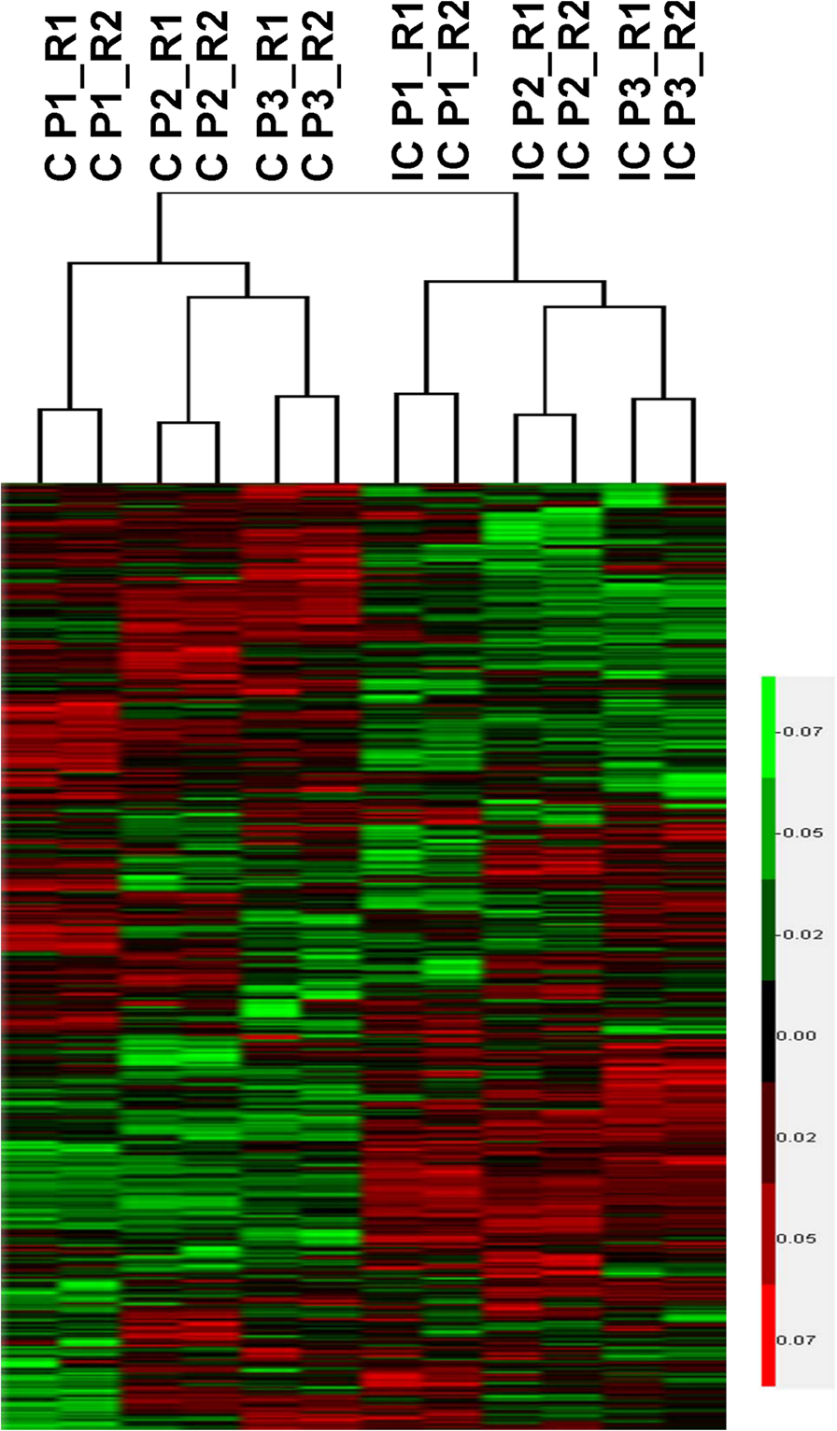
Hierarchical cluster analysis of all the proteins identified in different areas, biological replicates and technical replicates.

Among 46 recipient ewes, there were 39 ewes successful pregnant. Then we collected endometrium samples of these pregnant ewes at Day 17 of pregnancy (Figure 2). By LC-MS/MS proteomic analysis, we successfully detected 7459 and 7933 peptides in C areas and IC areas. After further protein identification, we identified 1740 and 1813 proteins in C areas and IC areas, relatively. Hierarchical clustering was performed based on the overall similarity of protein expression patterns of different areas and replicates. Results showed a striking separation of C and IC area samples into major opposing branches, implying that the endometrial proteomes of two areas are very distinct from each other. In addition, technical replicates in each pool were tightly clustered in the same branch, confirming the reliability of our detection system (Figure 3). The precision of quantitation between the technical replicates was evaluated by Pearson’s correlation coefficient as previous study [15]. As shown in Additional file 1: Table S1 and Additional file 2: Figure S1, we found an average PCC of 0.9871 for the protein level and 0.9401 for the peptide level. According to the criteria proposed in Waanders et al.’s study (0.87 and 0.98 for peptide and protein respectively) [16], the correlation values between technical replicates should be satisfying.

### Comparison of proteomic profile between C areas and IC areas

**Figure 4:**
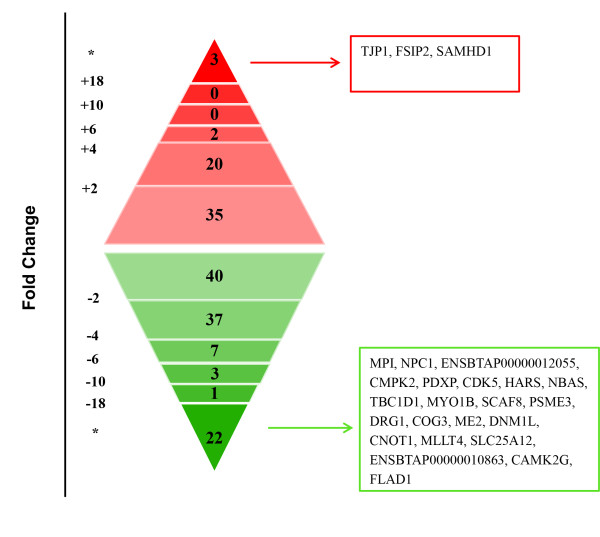
**Distribution of differentially expressed proteins with different fold change in the comparison between C and IC areas.** “*” means these proteins specifically expressed in C or IC areas.

By comparing proteomic profile between C areas and IC areas, we found 170 DEPs (*P* < 0.05). Among these DEPs, 60 proteins were up-regulated in C areas, and 110 proteins were up-regulated in IC areas. The most increased protein (fold change > 5) in C areas was GLANS (N-acetylgalactosamine-6-sulfatase precursor, 5.0-fold). In IC areas, the highest increased proteins (fold change > 5) in C areas included PIP4K2C (Phosphatidylinositol 5-phosphate 4-kinase type-2 gamma, 16.2-fold), PLIN4 (Uncharacterized protein, 6.6-fold), EML4 (echinoderm microtubule-associated protein-like 4, 6.6-fold) and ITGA1 (integrin, alpha 1, 6.1-fold). In addition, we detected 3 proteins specifically expressed in the C areas, and 22 proteins specifically expressed in the IC areas (Figure 4).

**Figure 5:**
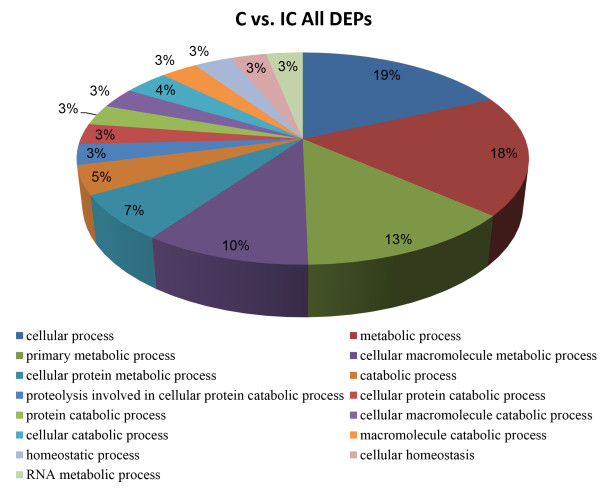
Function annotation clustering results with differentially expressed proteins between C and IC areas based on GO biological process.

To gain insight into different biological functions involved in implantation associated with C and IC areas, the 170 DEPs were analyzed using Functional Annotation Tool of DAVID Bioinformatics Resources 6.7. Finally, we identified 43 significant enriched Gene Ontology (GO) categories (*P* < 0.01) based on the major category of “biological process”, including “primary metabolic processes”, “cellular metabolic process”, “protein metabolic process”, “cell adhesion” and “multicellular organismal development” (Figure 5). The analysis also identified 9 significant enriched Kyoto Encyclopedia of Genes and Genomes (KEGG) pathways (*P* < 0.05), including “Focal adhesion”, “Regulation of actin cytoskeleton” and “ECM-receptor interaction” (Table 1).

**Table 1:** Significantly enriched KEGG pathways for DEPs between C and IC areas

**Pathway name**	**Count**	**Differentially expressed proteins**	***P*-value**
Focal adhesion	14	ACTG1,ACTN1,COL1A2,COL6A1,COL6A2,FLNA,ITGA1,LAMA4,LAMC1,MYLK,MYL9,COL6A3,TLN1,VCL	6.3E-6
ECM-receptor interaction	8	COL1A2,COL6A1,COL6A2,HSPG2,ITGA1,LAMA4,,LAMC1,COL6A3	2.0E-4
Pyruvate metabolism	4	**ALDH7A1**,**DLD**,LDHA,ME2	2.2E-2
Adherens junction	5	ACTG1,ACTN1,MLT4,**TJP1**,VCL	2.4E-2
Valine, leucine and isoleucine degradation	4	**HMGCL**,**ACAA1**,**ALDH7A1**,**DLD**	3.5E-2
Regulation of actin cytoskeleton	8	ACTG1,ACTN1,GSN,ITGA1,MYLK,MYL9,PIP4K2C,VCL	3.5E-2
Tight junction	6	ACTG1,ACTN1,MLT4,MYH11,MYL9,**TJP1**	4.9E-2

Proteins with higher expression level in C areas were shown in boldface; proteins with higher expression in IC areas were shown in lightface.

**Figure 6:**
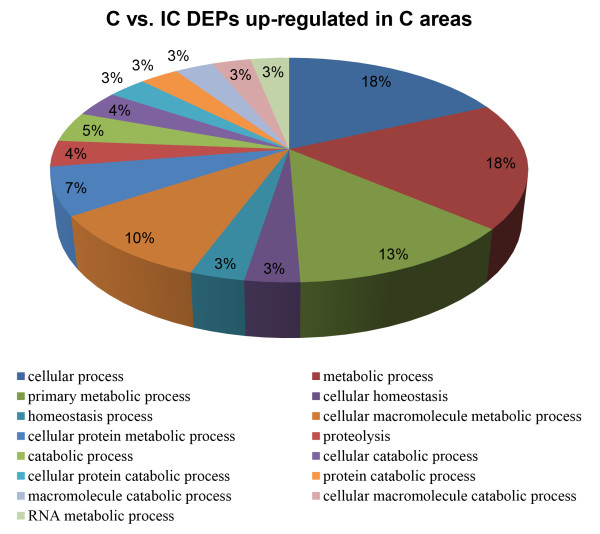
**Significantly enriched GO categories (*P*** **< 0.01) using differentially expressed proteins that were up-regulated in C areas.**

To better understand the structural and functional differences between these two areas, we divided all the DEPs into 2 groups: 60 DEPs that were up-regulated in C areas and 110 DEPs that were up-regulated in IC areas. These two groups of DEPs were then analyzed using DAVID platform respectively. For DEPs up-regulated in C areas, we identified 12 significant enriched GO terms (*P* < 0.01), based on the major category of “biological process”. “primary metabolic processes”, “cellular metabolic processes”, “cellular protein metabolic process” and “proteolysis involved in cellular protein catabolic process” were the most represented processes (Figure 6). KEGG pathway analysis just identified 1 significant pathway (*P* < 0.05), which is “Valine, leucine and isoleucine degradation”.

**Figure 7:**
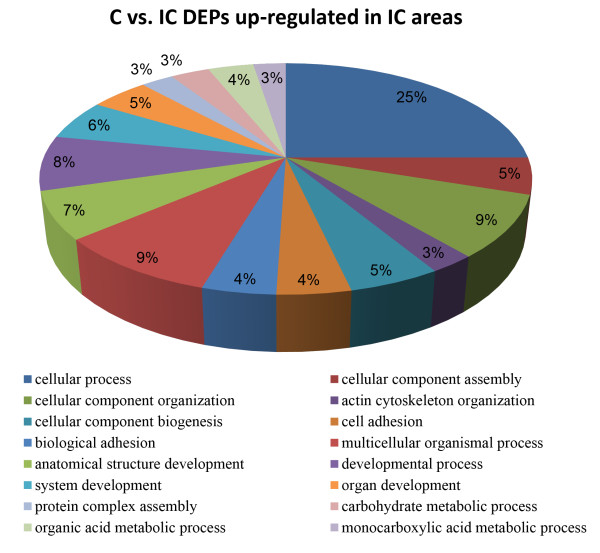
**Significantly enriched GO categories (*P*** **< 0.01) using differentially expressed proteins that were up-regulated in IC areas.**

For DEPs that were up-regulated in IC areas, we found 26 significant enriched GO categories (*P* < 0.01) based on the major category of “biological process”, including “cellular process”, “cellular component organization”, “actin cytoskeleton organization”, “multicellular organismal development” and “cell adhesion” (Figure 7). Our analysis also identified 7 significant enriched KEGG pathways (*P* < 0.05), which were major involved in cell adhesion and cell migration. These pathways included “Focal adhesion”, “ECM-receptor interaction” and “Tight junction” (Table 2).

**Table 2:** **Significantly enriched KEGG pathways (*P*** **< 0.05) using proteins with higher expression levels in IC areas**

**Pathway name**	**Count**	**Differentially expressed proteins**	***P*-value**
Focal adhesion	14	ACTG1,ACTN1,COL1A2,COL6A1,COL6A2,FLNA,ITGA1,LAMA4,LAMC1,MYLK,MYL9,COL6A3,TLN1,VCL	7.1E-8
ECM-receptor interaction	8	COL1A2,COL6A1,COL6A2,HSPG2,ITGA1,LAMA4,,LAMC1,COL6A3	1.8E-5
Regulation of actin cytoskeleton	8	ACTG1,ACTN1,GSN,ITGA1,MYLK,MYL9,PIP4K2C,VCL	5.2E-3
Arrhythmogenic right ventricular cardiomyopathy (ARVC)	4	ACTG1,ACTN1,DES,ITGA1	3.7E-2
Leukocyte transendothelial migration	5	ACTG1,ACTN1,MLT4,MYL9,VCL	3.7E-2
Adherens junction	4	ACTG1,ACTN1,MLT4,VCL	4.0E-2
Tight junction	5	ACTG1,ACTN1,MLT4,MYH11,MYL9	4.9E-2

Besides, we also used DAVID Bioinformatics Resources 6.7 to analyze the specifically expressed proteins in IC areas. Finally, we found these proteins were significant enriched (*P* < 0.01) in two GO categories, “organic acid metabolic process” and “regulation of cell communication”.

## Discussion

The endometrium of sheep consists of C and IC areas, both of which are essential for the establishment of implantation and maintenance of pregnancy. In order to study the different contribution of C areas and IC areas to the implantation, we used LC-MS/MS technique to profile the proteome of these two areas separately at Day 17 of pregnancy. By comparing the proteomic profiles between C areas and IC areas, we found many biologically meaningful DEPs. Based on these DEPs, bioinformatics analysis revealed the different biological functions between these two areas on protein level. These important functional differences were mainly associated with growth and remodeling of endometrial tissue, cell adhesion and protein transport. Our results provided data references and materials for further studies. It should be noted that these two regions contain varying amounts of different cell types, including LE, GE, stroma, blood and lymph vessels, and immune cells, and the gene or protein expression pattern shows temporal and spatial changes in endometrium [19], Therefore, previous study have profiled the endometrial transcriptome of individual cell populations using laser capture microdissection [20]. However, in the most of the studies concerning the endometrium during establishment of pregnancy in ruminants, endometrium was sampled as an integrated tissue layer for studying the endometrium involved mechanisms of pregnancy establishment or maternal-fetal interaction [2,4,9,21,22], In addition, due to the relative large requirements of sample amount for LC-MS/MS analysis, profiling the proteome of a single cell type in endometrium is impractical. Therefore, in our study, a wildly accepted strategy was used to establish the sample pools of endometrium with different cell types. The collection of tissue samples were performed strictly according to the method reported in the previous studies [4,9]. We did not provide the detailed proteomic difference between the distinct cell types in C and IC areas, however, this limitation did not compromise the significance of our study.

### Growth and remodeling of endometrial tissue

During implantation, ovine endometrium always undergoes extensive and sufficient structural modification and tissue growth, in order to accommodate and support rapid conceptus development and growth [3]. The series of biological processes were referred to as endometrial remodeling. It has been demonstrated that appropriate endometrial remodeling is essential for successful implantation during early pregnancy [3]. Given structural and functional differences between C and IC areas, they have different ways in remodeling.

As C areas are major responsible for conceptus attachment and placentation, the substantial remodeling in these areas are crucial for successful implantation. In the present study, GO category “proteolysis” (PSMB3, NEDD8, UCHL3 and USP34) was significantly over-represented in C areas. Among these proteins, PSMB3 is a member of proteasome. The proteasome is an ATP-dependent, multi-subunit, multi-catalytic protease complex that is involved in recognizing and degrading ubiquitinated proteins in most non-lysosomal pathway of protein degradation in eukaryotic cells [23]. It has been reported that proteasome participate in tissue remodeling of the conceptus and endometrium during pregnancy in human and mouse [24]. Up to now, however, only few studies have reported the specific functions of proteasome in ovine endometrium during early pregnancy. Our results indicated that proteasome are likely to participate in the tissue remodeling of endometrial C areas through degrading proteins during implantation. NEDD8, UCHL3 and USP4 are highly associated with ubiquitination. Many previous studies have demonstrated that ubiquitination-related proteins are expressed in cells of uterus in many species, and these proteins play vital roles in endometrial remodeling and placentation during pregnancy [25]. The up-regulation of above mentioned proteins in C areas indicated that C areas undergo extensive and substantial tissue remodeling through ubiquitin-proteasome system (UPS) based proteolysis during the peri-implantation period. Besides, we found the expression of TAGLN was drastically increased in C areas (fold-change = 2.15). TAGLN is constitutively expressed in pregnant uterus, and previously regarded as a biomarker for arterial vessel remodeling in uterine tissue [14]. The up-regulation of TAGLN in C areas implied that vascular remodeling may play crucial roles in endometrial remodeling preparing for implantation. As C areas are key tissues for placentation, the increased vascular remodeling in C areas would also be essential for ensuring normal placental function.

Unlike C areas, there are many endometrial glands localized in IC areas. During the peri-implantation period, these endometrial glands of the IC areas experience substantially growth in length and width, to ensure normal synthesis of nutrition to support the development of conceptus [3]. Thus, we can conclude that IC areas also underwent a considerable remodeling process in the form of proliferation and morphogenesis of the intercaruncular endometrial glands. In our study, many DEPs that were up-regulated in the IC areas were significantly enriched in GO categories and KEGG pathways functionally associated with endometrial gland morphogenesis. Most of these proteins, including ACTA2, ACTG1, ACTN1, MYLK and MYL9, are the structural constituents of cytoskeleton, which is essential for morphologic changes of tissues and cells [26]. In these proteins, ACTA2, ACTG1 and ACTN1 belong to actin family of proteins. It has been reported that actin plays important roles in cell motility, structure, and integrity [27]. MYLK and MYL9 belong to myosin, which is responsible for muscular movement. Myosin may participate in cell motility and contraction in endometrial glands. The up-regulation of all above proteins in IC areas indicated that endometrial glands of IC areas undergo extensive changes in cell morphology during the peri-implantation period, which may prepare for increased nutrient production. In addition, many proteins specifically expressed in IC areas were enriched in GO categories related to cell proliferation, including CAMK2G, CDK5, CMPK2 and HARS. CDK5 belongs to cyclin dependent kinases, which is involved in regulating cell proliferation and differentiation, as well as apoptosis. These specifically expressed proteins made us to speculate that IC areas underwent an increased cell proliferation compared with C areas during the peri-implantation period. Together our findings in IC areas, is presumable that both cytoskeleton remodeling and cell proliferation play crucial roles in endometrial gland morphogenesis.

Based on all above results, we provided proteomics evidence that both C areas and IC areas were characterized by a considerable remodeling process, which was a preparation for successful implantation. However, these two areas participated in endometrial remodeling through different patterns. The tissue remodeling in C areas was mainly through proteasome-dependent proteolysis and vascularization. Unlike C areas, IC areas underwent endometrial gland morphogenesis through accelerating cytoskeleton remodeling and cell proliferation.

### Cell adhesion

Cell adhesion is one of the most important physiological processes involved in implantation. Abnormal cell adhesion may lead to an early pregnancy failure. In the present study, many DEPs were functionally associated with cell adhesion.

We found two proteins related to cell adhesion, TJP1 and POSTN, were specifically expressed in C areas. TJP1 belongs to tight junction proteins. Tight junctions are always located on the plasma membrane, and maintain cell polarity, cell-cell contact, and cell adhesion [28]. POSTN, an important extracellular matrix (ECM) protein, plays an important role in facilitating cell adhesion and migration. It has been reported that POSTN may induce PI3K/AKT signaling to stimulate attachment and migration of ovine trophectoderm (oTr) cells during early pregnancy in ewes. Besides, POSTN may influence placentation and placental functions [27]. Therefore, our results indicated that cell adhesion, as an very important and specialized physiological process in the C areas during the peri-implantation period, were ensured by specifically expression of related proteins.

We also found many DEPs up-regulated in the IC areas were significantly enriched in GO categories and KEGG pathways related to cell adhesion. GO category “cell junction” (TLN1, TNS1 and VCL) was overrepresented in the IC areas. These three proteins are important cytoskeleton proteins. They play crucial roles in integrin-mediated cell adhesion, migration and proliferation by activating integrin signaling pathway [29,30]. Besides, KEGG pathways “ECM-receptor interaction” and “focal adhesion” (COL1A2, COL6A2, LAMA4, LAMC1 and ITGA1) were over-represented in IC areas. Among these proteins, ITGA1 was dramatically increased in IC areas (fold-change = 6.07). ITGA is one kind of integrins. Integrins are a family of cell adhesion molecules that have now been largely accepted as biomarkers of uterine receptivity [31]. During implantation, integrins are responsible for mediating adhesion between maternal and embryonic epithelium [32,33]. COL1A2, COL6A2, LAMA4, LAMC1 belong to ECM. ECM participates in cell adhesion mainly through binding to cell adhesion molecules. These results indicated that contrast with traditional knowledge, IC areas may also played important roles in cell adhesion through many biological processes and pathways, such as cell junction, ECM-receptor interaction and focal adhesion.

Taken together, we found both C areas and IC areas participate in cell adhesion during the peri-implantation period. Considering that C areas are the major sites for conceptus attachment, most previous studies related to cell adhesion during implantation focused on these areas. However, our results indicated that IC areas also played important roles in cell adhesion. One possible explanation was that endometrial glands of IC areas may synthesize a lot of proteins related to cell adhesion, such as cytoskeletal proteins, integrins, collagens and laminins, and then secrete them into C areas or uterine lumen to participate in cell adhesion. Thus, we inferred that C areas mediate cell adhesion during implantation in collaboration with IC areas.

### Protein transport

In ruminants, the growth and development of the conceptus depend largely on endometrial glands in IC areas. During early pregnancy, these glands synthesize, secrete or transport various enzymes, growth factors, cytokines, hormones, transport proteins and adhesion molecules, collectively referred as histotroph. Gray et al. reported that secretions of endometrial glands were required for peri-implantation conceptus survival and development, and uterine gland knockout ewes (UGKO) were unable to support pregnancy up to Day 25 of pregnancy [34]. Besides, many studies demonstrated that some secretions of endometrial glands are the primary regulators of fetal-maternal dialogues.

In the present study, GO category “protein transport” (HSPA8, SYPL1 and TEMD10) was significantly over-represented in IC areas. HSPA8 functions as an ATPase during transport of membrane proteins through the cell. SYPL1 is associated with GLUT4-cotaining vesicles [35]. GLUT4 has been found in the human placenta, which is mainly responsible for controlling uptake of glucose into cells [36]. Therefore, the reduced expression of GLUT4 in endometrial cells may lead to metabolic defects, which has adverse effect on implantation. TMED10 is type I membrane protein, which is localized to the plasma membrane and golgi cisternae and is involved in vesicular protein trafficking.

Together these results, we found protein transport is an important physiological process associated with IC areas. Compared to C areas, protein transport played a more important role in IC areas. Abnormal protein transport may have adverse effects on secretion function of endometrial glands, leading to implantation failure.

## Conclusion

We established a relatively comprehensive and detailed protein database of ovine endometrium by using LC-MS/MS technique, providing data references for further researches. By comparing proteomic profiles between C and IC areas, we detected 170 DEPs. Subsequent bioinformatics analysis indicated these DEPs were mainly involved in a lot of important physiological processes, including growth and remodeling of endometrial tissue, cell adhesion and protein transport. Based on these results, we provided proteomics evidence that both C areas and IC areas were characterized by a considerable remodeling process, which was a preparation for successful implantation. In addition, we found that C areas could mediate cell adhesion during implantation in collaboration with IC areas. Lastly, results showed that protein transport was an important physiological process associated with endometrial glands, which played a more important role in IC areas relatively.

## Abbreviations

C: Caruncles; IC: Intercaruncular areas; DEPs: Differentially expressed proteins; P4: Progesterone; IFNT: Interferon tau; LC-MS/MS: Liquid chromatography-tandem mass spectrometry; CIDR: Controlled intra-vaginal drug release; AI: Artificial insemination; TCA: Trichloroacetic acid; DTT: Dithiothreitol; PMSF: Phenylmethanesulfonyl fluoride; EDTA: Ethylenediaminetetraacetic acid; IAM: Iodoacetamide; ACN: Acetonitrile; FA: Formic acid; nano-HPLC: Nano-high performance liquid chromatography; UPS: Ubiquitin-proteasome system; oTr: Ovine trophectoderm; UGKO: Uterine gland knockout.

## Competing interests

The authors declare that they have no competing interests.

## Authors’ contributions

YW and CW analyzed the data and drafted the manuscript. LA designed the study and revised the manuscript. ZCH preformed the statistical analysis. KM, HCZ, RW and MG participated in the collection of samples. JHT and ZHW participated in the design of the study. All authors have read and approved the final manuscript.

## Supplementary Material

Additional file 1: Table S1 Summary of Pearson’s correlation coefficient (R) between technical replicates in each pool of different groups. Click here for file

Additional file 2: Figure S1 Pearson correlation coefficient (PCC) of protein level between technique replicates in each pool of different groups. The reliability of protein quantitation between the technical replicates was evaluated by PCC. Click here for file
