# Supplementary material for: Comparative analysis of proteomic profiles between endometrial caruncular and intercaruncular areas in ewes during the peri-implantation period
Source: J Anim Sci Biotechnol. 2013 Oct 5;4(1):39. doi: 10.1186/2049-1891-4-39 (PMC3892124; doi:10.1186/2049-1891-4-39)
Supplement: Additional file 1: Table S1 — Summary of Pearson’s correlation coefficient (R) between technical replicates in each pool of different groups. [file 2049-1891-4-39-S1.docx]

**Supplemental Table S1**: Summary of Pearson’s correlation coefficient (R) between technical replicates in each pool of different groups.

| **Group** | **Biological replicates** | **Technical replicates** | **R (peptide level)** | **R (protein level)** |
| --- | --- | --- | --- | --- |
| **C areas** | Pool 1 | rep1_rep2 | 0.9254 | 0.9833 |
|  | Pool 2 | rep1_rep3 | 0.9613 | 0.9861 |
|  | Pool 3 | rep2_rep3 | 0.9465 | 0.9777 |
| **IC areas** | Pool 1 | rep1_rep2 | 0.9339 | 0.9923 |
|  | Pool 2 | rep1_rep3 | 0.9119 | 0.9967 |
|  | Pool 3 | rep2_rep3 | 0.9614 | 0.9866 |
|  |  | Average R | 0.9401 | 0.9871 |
|  |  | Range of R values | 0.9254-0.9614 | 0.9833-0.9967 |
