# Supplementary figures and images for: Comparative analysis of proteomic profiles between endometrial caruncular and intercaruncular areas in ewes during the peri-implantation period
Source: J Anim Sci Biotechnol. 2013 Oct 5;4(1):39. doi: 10.1186/2049-1891-4-39 (PMC3892124; doi:10.1186/2049-1891-4-39)

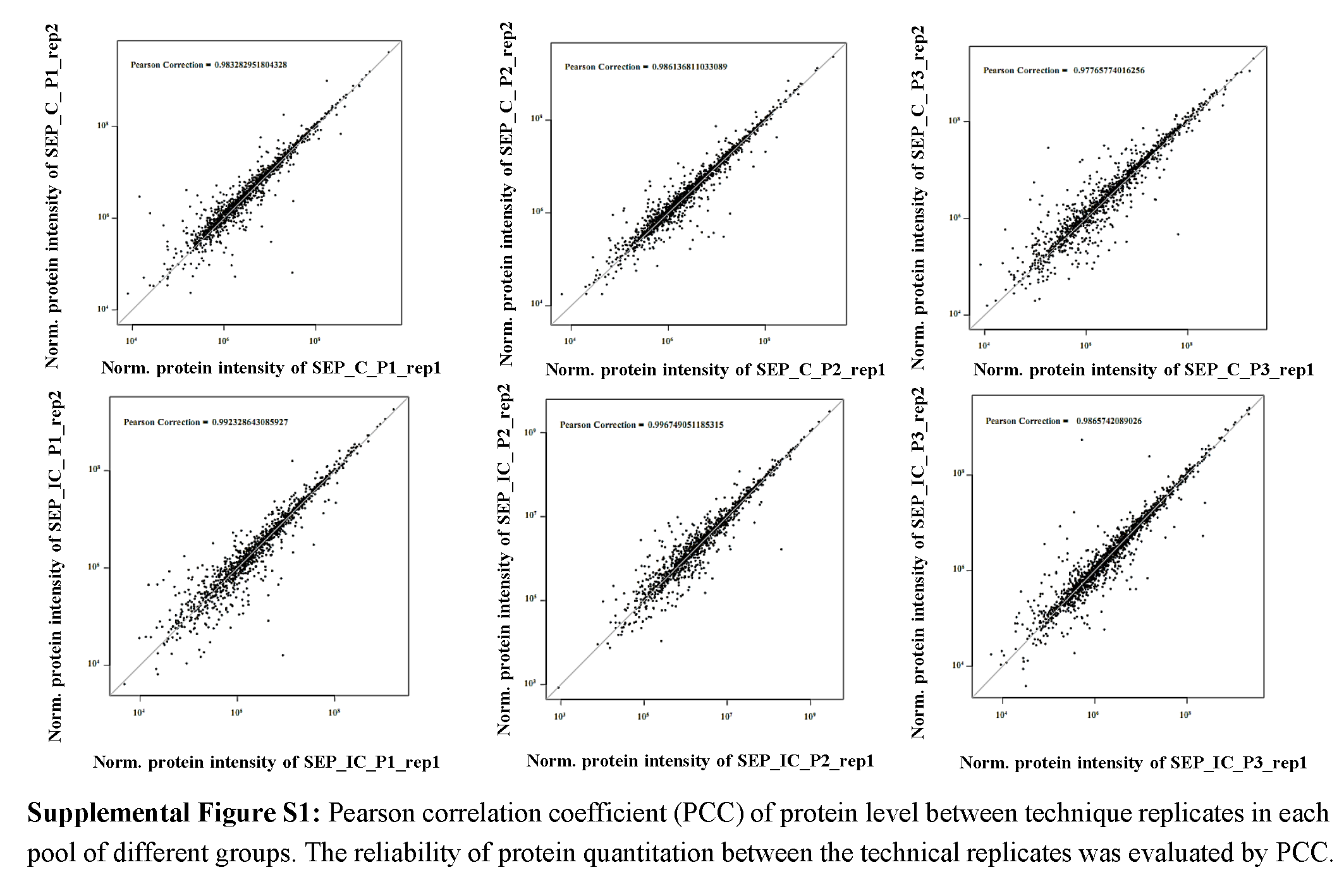

Supplement: Additional file 2: Figure S1 — Pearson correlation coefficient (PCC) of protein level between technique replicates in each pool of different groups. The reliability of protein quantitation between the technical replicates was evaluated by PCC. [file 2049-1891-4-39-S2.tiff]
